# Nurses' Perspectives on Postpartum Pain Management

**DOI:** 10.1089/whr.2021.0104

**Published:** 2022-03-04

**Authors:** Benjamin R. Loomis, Lynn M. Yee, Lauren Hayes, Nevert Badreldin

**Affiliations:** ^1^ Department of Obstetrics and Gynecology, Northwestern University Feinberg School of Medicine, Chicago, Illinois, USA.; ^2^ Division of Maternal-Fetal Medicine, Department of Obstetrics and Gynecology, Northwestern University Feinberg School of Medicine, Chicago, Illinois, USA.; ^3^ Department of Obstetrics and Gynecology, Northwestern Memorial Hospital, Chicago, Illinois, USA.

**Keywords:** nursing care, opioid, pain management, postpartum care, survey research

## Abstract

**Introduction:**

There is variation in postpartum opioid use by prescriber characteristics that cannot be explained by patient or birth factors. Thus, our objective was to evaluate nursing training, clinical practices, and perspectives on opioid use for postpartum pain management.

**Materials and Methods:**

In this survey study, postpartum bedside nurses at a single, large academic center were asked about training, factors influencing clinical decisions, and viewpoints regarding pain management and opioid use. Findings were summarized using descriptive analyses.

**Results:**

A total of 92 nurses completed the survey. A majority (77%) reported having received some formal training on opioid use for pain management. About a quarter (25.7%) felt their training was not adequate. Regarding clinical practices, the majority (71% and 70%, respectively) reported that “routine habit” and “patient preference” most influenced the type and amount of pain medication they administered. Finally, nurses' perspectives on pain management demonstrated a wide range of beliefs. Most nurses strongly agreed with the importance of maximizing nonopioid pain medication before opioid administration. The majority agreed that patient-reported pain score is important to consider when deciding to administer opioids. Conversely, most nurses disagreed that patients should be encouraged to endure as much pain as possible before using an opioid. Similarly, beliefs about the reliability of use of vital signs in assessing pain intensity varied widely.

**Conclusions:**

Bedside nurses rely on routine habits, patient preference, and patient-reported pain score when administering opioids for postpartum pain management. Increased training opportunities to improve consistency and standardization of opioid administration may be beneficial.

## Introduction

Hospitalizations are a significant source of opioid exposure for patients, with over half of all nonsurgical hospitalizations resulting in inpatient opioid use.^1^ This exposure puts patients at risk for opioid-related adverse events, persistent opioid use, and resulting complications.^1,2^ Despite recommendations and guidelines by many leading organizations to encourage more prudent opioid use and prescribing,^3–5^ opioid-related deaths remain high.

The trend of inpatient opioid use is ubiquitous, and the postpartum setting is no different. Opioids are commonly used in the inpatient postpartum setting and prescribed upon discharge.^6–8^ In light of labor and delivery remaining a leading reason for hospitalization in the United States, understanding the factors affecting opioid use in this setting is critical to combat excess postpartum opioid use.

Recent work has elucidated prescriber-level factors that influence opioid use in the postpartum setting. For example, care by an advanced practitioner, including certified nurse midwives, nurse practitioners, attending physician providers, or trainee physicians, is associated with decreased odds of postpartum opioid use by almost 50%.^7^ Trainee physicians were also significantly less likely to prescribe a high amount of opioids at postpartum hospital discharge when compared with attending physicians (adjusted odds ratio, 0.01; 95% confidence interval, 0.00–0.36).^9,10^

Nonclinical factors may also influence opioid prescribing. For example, Hispanic and non-Hispanic Black patients had greater odds of reporting higher pain scores than their non-Hispanic White counterparts, but received less pain medication.^10^ While it is clear that many factors affect postpartum opioid use, the impact of bedside nurses has been understudied in this setting.

As the health care team member who interfaces the most with patients, a bedside nurse is an essential aspect of care for postpartum patients. Oral analgesic orders, specifically opioid orders, are often entered as *pro re nata* (PRN), therefore giving nursing staff autonomy to assess patients, counsel them regarding available pain management options, and administer medications. Previous work has addressed nurses' perspectives on pain management in patients hospitalized for heart failure.^11^

Nurses reported awareness of the need for individualization in pain management and inpatient opioid use, but they voiced a desire for improvements in training and development of evidence-based protocols. These findings have been echoed in other nonobstetrical populations.^12,13^ Similar research is lacking in the postpartum setting. Thus, the objectives of this study were to evaluate nurses' training, clinical practices, and perspectives on opioid pain management in the inpatient postpartum setting.

## Materials and Methods

This was a survey study of all postpartum bedside nurses at a single, large, academic tertiary care center. Surveys were administered from July to November 2020. All postpartum registered nurses and registered nurse managers who regularly provided care on the postpartum unit were eligible to participate. Nurses who floated from other units or were temporarily assigned to this unit for other reasons were not eligible. All nurses were over 18 years of age and English speaking.

This study was conducted at a tertiary care institution that performs more than 11,000 deliveries per year. The patients are racially and ethnically diverse and have diverse socioeconomic backgrounds.^8^ They are cared for by a diverse group of obstetrical care providers, including trainees, advanced practitioners, and obstetrics and gynecology attending physicians. Following birth, patients are transferred to the postpartum unit where they typically spend 2–4 days, depending on the mode of delivery.

There are 105 single-occupancy postpartum beds over 4 floors, and postpartum rooms are assigned based on availability and staffing considerations. Patient and neonate (when applicable) dyads are cared for by bedside nursing staff at a ratio of 1:4 unless clinical circumstances warrant a lower ratio. Each nurse maintains their census of patients for the duration of a shift. Typical postpartum nursing shifts are 12 hours.

Following childbirth, the delivering provider places analgesic orders for postpartum care. During the study period, available standard order sets included scheduled ibuprofen every 6 hours and PRN acetaminophen every 6 hours. Order sets following vaginal birth do not include an option to prescribe opioids; if opioids are desired, they require a separate order be written and updated by the prescriber. Postpartum cesarean birth order sets include the option for oxycodone 5 mg PRN every 4 hours for pain.

All medications, including opioids, are administered by the bedside nursing staff. Bedside nursing staff assess pain and record pain scores at scheduled intervals of 8 hours, before and after pain medications are received, and upon discharge; patients are asked to report their current perception of pain, with 0 indicating no pain and 10 indicating the worst pain imaginable. In addition, bedside nurses counsel and administer PRN analgesia medications as needed. There was no standardized or formal nursing education regarding opioid administration that occurred during this study period.

No validated surveys specific to perspectives on postpartum pain management were available and therefore survey questions were derived based on clinical expertise, review of available literature,^14^ and discussion among the study team. Survey questions focused on several aspects of nurse experience and practices (Supplementary Data). The first section focused on pain management training and, when applicable, assessing the amount, focus, and format of the training.

The second section assessed participants' current clinical practices and factors that influence these practices. This included asking participants the frequency with which they recommended various pain management methods (on a scale of 1 [never] to 5 [always]). The final section was designed to elicit nurses' perspectives on postpartum pain management by asking participants to respond on a Likert scale from 1 (strongly disagree) to 5 (strongly agree) to several statements centered on clinical management of pain in the postpartum setting.

Results were reported descriptively or aggregated and reported as median and interquartile range (IQR). Before distribution, the survey was assessed for clarity by three experienced bedside nurses, who were thereafter not eligible for participation.

A list of participants was compiled using an institutional email listserv of all eligible registered nursing staff with review of the eligible list conducted *via* nursing management staff. Participants received the study invitation *via* email with periodic reminders sent over the course of the study period. They completed a 10-minute online survey (described below) regarding their formal training, clinical practices, and perspectives on postpartum pain management and opioid use.

Data were entered into Research Electronic Data Capture (REDCap),^15^ a secure, online data management platform. No identifying information was collected in the survey and individual responses were deidentified. Participants received $5 gift cards following completion of the survey. Survey findings were summarized using descriptive analysis using Microsoft Excel. As all responses were deidentified and no protected health information was collected, the Northwestern University Institutional Review Board provided a waiver for this study and it did not require consent.

## Results

Of 166 nurses eligible for participation, 92 (55.4%) completed the survey between July and November 2020. Demographic information of respondents is summarized in Table 1. Almost all participants identified as female (*n* = 91; 98.9%), non-Hispanic (*n* = 85; 92.4%), and White (*n* = 78; 84.8%). The majority work primarily in the postpartum inpatient clinical setting (*n* = 89; 96.7%), with few identifying the newborn nursery (*n* = 9; 9.8%) and labor and delivery (*n* = 6; 6.5%) as additional clinical settings. The median number of years of clinical experience was 4 years (range, 0–40; IQR, 2–7) and the median number of hours worked in clinical care per month was 144 hours (range, 4–320; IQR, 97–144).

**Table 1.** Demographics, Work, and Education

**Characteristics**	***N* = 92**
Age, years	28 (22–65)
Identifying gender	
Female	91 (98.9)
Male	1 (1.1)
Ethnicity	
Non-Hispanic	85 (92.4)
Hispanic	6 (6.5)
Race	
Asian	7 (7.6)
Black or African American	3 (3.3)
White	78 (84.8)
Other	1 (1.25)
Clinical work location	
Labor and delivery	6 (6.5)
Newborn nursery	9 (9.8)
Postpartum	89 (96.7)
Clinical care (hours/month)	144 (4–320)
Administration/teaching (hours/month)	0 (0–144)
Years in practice	4 (0–40)
Education	
Bachelor's	77 (84.7)
Master's	13 (14.1)
Doctorate	2 (2.2)

Data provided as median (interquartile range) or *n* (%) as appropriate.

Nurses' previous training in both general and postpartum pain management and opioid-specific pain management is shown in Table 2. With regard to general pain management, 91.2% (*n* = 83) reported receiving any form of pain management training. Online modules were the most common medium of training received (*n* = 68; 74.7%), followed by coursework done for degree attainment (*n* = 44; 48.4%) and education-centered emails from leadership (*n* = 34; 37.4%).

**Table 2.** Postpartum Nurses' Training in Postpartum Pain Management

**Pain management**	***n***	**General clinical setting (%)**	***n***	**Postpartum setting (%)**
Any form	83	90.1	83	90.1
Online module	68	74.7	51	56.0
Degree course work	44	48.4	13	14.3
Leadership emails	34	37.4	30	33.0
In-person lectures	30	33.0	19	20.9
Colleague mentorship	27	29.7	23	25.3
Other	1	1.1	4	4.4

**Opioid pain management**				
Any form	74	81.3	74	81.3
Online module	45	49.5	35	38.5
Degree course work	22	24.2	8	8.8
Leadership emails	37	40.7	34	37.4
In-person lectures	22	24.2	14	15.4
Colleague mentorship	20	22.0	22	24.2
Other	3	3.3	9	9.9

Pain management training that was specific to the postpartum setting followed a similar pattern, with online modules being the most common training medium (*n* = 51; 56%). The median number of hours of training received was 2 hours (range, 0–50; IQR, 1–4). About two-thirds of total respondents (*n* = 63; 68.5%) stated that training provided the necessary knowledge for their clinical practice.

Regarding training specific to opioid management, a majority (*n* = 74; 81.3%) reported having received some form of training on opioid use for pain management (Table 2). Training similarly was most commonly received in the form of online modules for opioid-specific general (*n* = 45; 49.5%) and postpartum pain management (*n* = 35; 38.5%). The median number of hours of training received was 2 (IQR, 1–4), and 74.3% (*n* = 55) of respondents who received training felt that it provided the necessary knowledge.

**FIG. 1.**
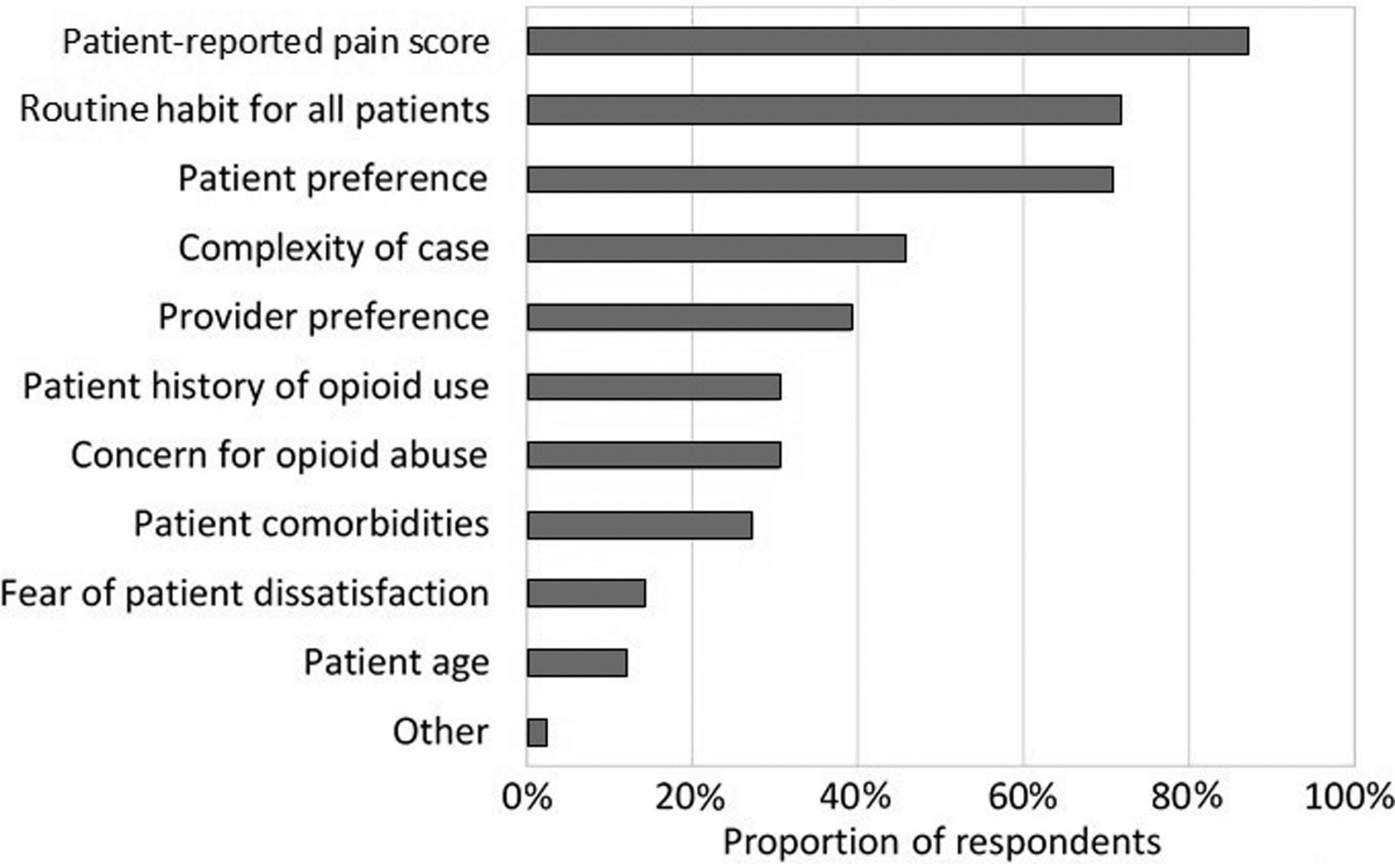
Factors influencing opioid administration. Nurses' responses to the question, “What most affects the choice of pain medication type and amount utilized in postpartum care?” Bars represent the percentage of respondents; respondents could choose multiple responses.

Patient-reported pain score (*n* = 80; 87%), routine habit (*n* = 66; 71.7%), and patient preference (*n* = 65; 70.7%) were the most influential factors regarding the nursing choice of pain medication type and amount (Fig. 1). Respondents were less influenced by fear of patient dissatisfaction and concern for opioid abuse (14.1% and 30.4%, respectively).

Participants reported a median patient pain score of 7 of 10 (range, 4–9; IQR, 6–7) as the point they would recommend an opioid for pain management. The majority (*n* = 87; 95%) felt that opioid use is necessary in greater than half of all patients for pain management following a cesarean birth, whereas only 5% felt similarly regarding vaginal birth.

**FIG. 2.**
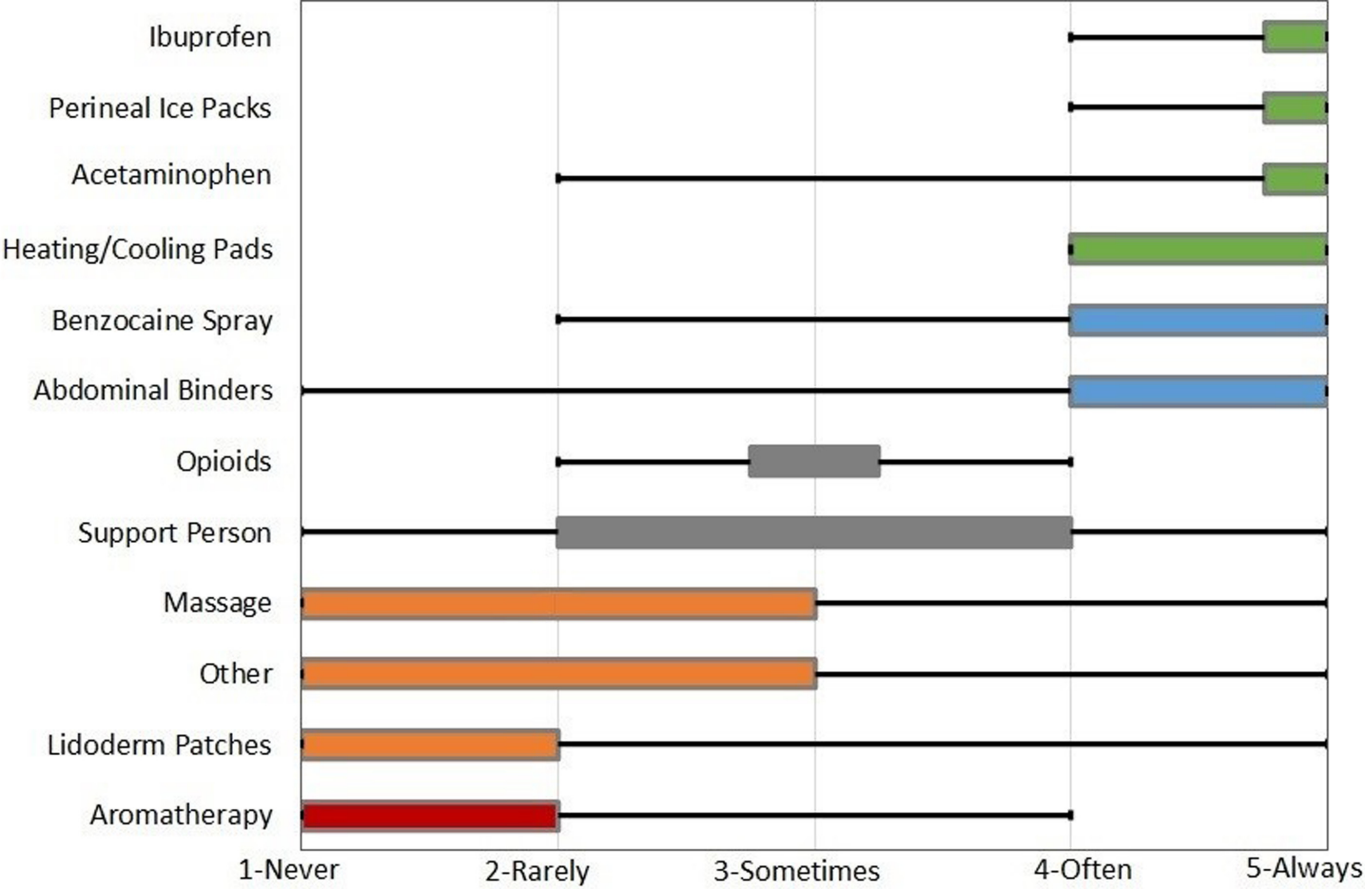
Pain management method recommendation frequency. Figure displays the median, IQR, and range. Medians are represented by location of diamond. Left side of the box represents the lower quartile, and right side represents the upper quartile. Small bar represents upper and lower quartiles being the same. Lower whisker to upper whisker represents the range. IQR, interquartile range.

Next, regarding pain management methods (Fig. 2), a wide range of practices were recommended by participants. On average, participants reported that they always (median = 5) recommend ibuprofen, perineal ice packs, and acetaminophen; sometimes (median = 3) recommended opioids; rarely (median = 2) recommended massage and Lidoderm patches; and *never* (median = 1) recommend aromatherapy. All participants reported reviewing available pain management options with patients at the time of discharge; however, only 9.8% of nurses stated that they specifically reviewed safe opioid medication storage or disposal methods.

**FIG. 3.**
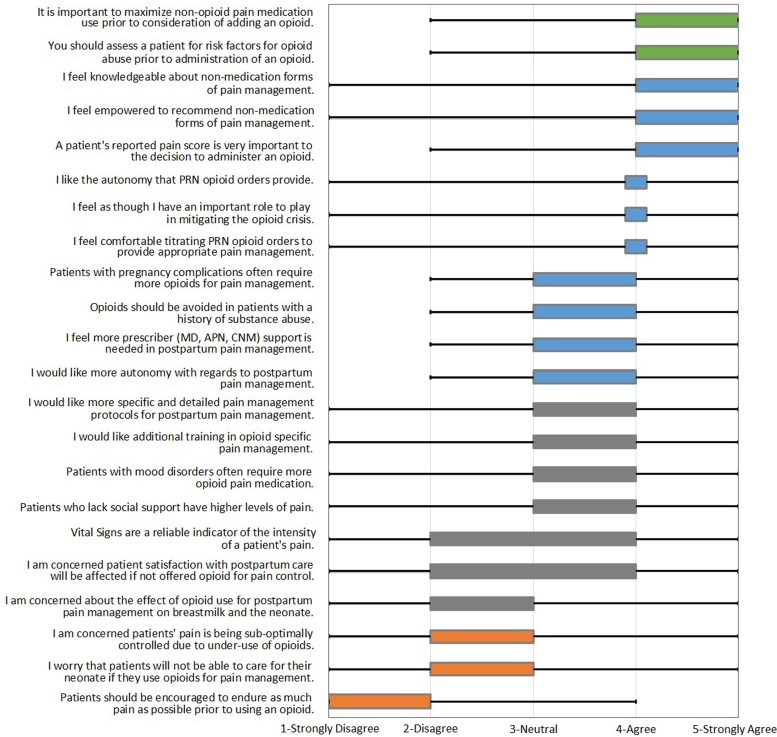
Perspectives on pain management practice. Figure displays the median, IQR, and range. Medians are represented by location of diamond. Left side of the box represents the lower quartile, and right side represents the upper quartile. Small bar represents upper and lower quartiles being the same. Lower whisker to upper whisker represents the range.

Finally, perspectives on pain management were elicited by asking participants their views on several statements centered on clinical management of pain in the postpartum setting (Fig. 3). On average, participants strongly agreed (median = 5) with the following statements:

- It is important to maximize nonopioid pain medication use (*e.g.*, ibuprofen and acetaminophen) before consideration of adding an opioid.
- You should assess a patient for risk factors for opioid abuse before administration of an opioid.

Conversely, on average, most participants disagreed (median 2) with the following statements:

- I am concerned patients' pain is being suboptimally controlled due to underuse of opioids.
- I worry that patients will not be able to care for their neonate if they use opioids for pain management.

## Discussion

Our study aimed to better understand nursing practices with regard to postpartum pain management. We demonstrate that bedside nurses rely on routine habits, patient preference, and patient-reported pain score when administering opioids. They often utilize nonopioid pain medications, such as acetaminophen and ibuprofen, but less commonly utilize nonpharmacologic pain management strategies.

Additionally, this survey demonstrated that while nursing staff enjoy the autonomy of PRN prescribing, they desire more prescriber support and training on opioid use. As members of the care team who interface among the most with postpartum patients, bedside nurses serve an essential role in pain management and patient opioid utilization and are critical to consider when implementing pain management guidelines and protocols.

Implementation of training on opioid use has been shown to decrease the amount of opioids prescribed.^16–18^ For example, a recent quality improvement study that aimed to reduce opioid overprescribing after common general surgical operations demonstrated a decrease in opioid prescribing following implementation of formal prescriber educational interventions and feedback on prescribing habits.^17^ Following implementation of interventions such as department-wide grand rounds, case-based conferences, and dedicated didactic sessions, there was an average decrease in the number of acetaminophen–hydrocodone pills prescribed, by 26 tablets (*p* < 0.001).

In another study of emergency department prescribers, feedback sessions, during which prescribers were shown their own opioid prescribing patterns in relation to those of their deidentified peers, resulted in a significant decrease in total opioid prescribing rates of about 3%.^18^ Notably, available interventions primarily focus on prescriber modification, and interventions that include nursing staff are considerably more limited. In our study, 81.3% of participating nurses reported receiving opioid-specific pain management training at any point in their career.

However, of those who did receive training, the median number of hours of cumulative training was only 2. Training modality was widely variable and the best format for future training still needs to be assessed. Particularly given the prior research suggesting the potential improvements in care that may occur after educational interventions, additional training opportunities for nurses in pain management and opioid use may be an important strategy for improved, evidence-based opioid use postpartum.

In this survey, nurses supported maximizing nonopioid analgesics, such as acetaminophen and ibuprofen, before opioid administration. Indeed, research on postpartum pain management has shown that increased use of acetaminophen is independently associated with decreased odds of opioid use.^6^ In particular, the combination of scheduled acetaminophen and ibuprofen has been shown to offer effective postpartum analgesia.^19^

As such, clinical consensus publications from the American College of Obstetricians and Gynecologists (ACOG) have recommended that obstetrician–gynecologists and other obstetric care professionals should use a stepwise multimodal approach to postpartum pain management. Specifically, ACOG recommends the use of a combination of agents with different mechanisms of action, starting with nonopioid analgesics such as acetaminophen and nonsteroidal anti-inflammatory drugs and escalating as necessary.^20^ In this survey, nurses' perspectives in this regard are concordant with current guidelines.

Research that implemented postpartum pain management protocols of scheduled acetaminophen and ibuprofen has shown a significant reduction in utilization of opioids postpartum.^21–23^ In one prospective study of more than 200 postpartum people, it was found that a regimen of alternating scheduled acetaminophen and ibuprofen resulted in a significant decrease in postpartum opioid use, from 143.2 to 105.8 morphine milligram equivalents (MMEs) in cesarean births and 32.8 to 26.1 MMEs in vaginal births.^2^

Additionally, it was found that nurses specifically played a key role in implementing this change and educating postpartum patients about analgesic options. Partnering with nurses to develop specific protocols for the use of nonopioid analgesics before opioid use has the potential to significantly decrease postpartum opioid use.

Nurses in this survey reported the importance of nonpharmacologic methods of pain management postpartum, such as the use of abdominal binders and perineal ice packs, and reported frequent use of these methods. In contrast, other nonpharmacologic methods such as massage, support persons, and aromatherapy were rarely recommended by nursing staff despite nurses reporting feeling knowledgeable about these methods and feeling empowered to utilize them. Thus, further training in these methods may be necessary to increase utilization.

Aromatherapy has been shown to offer benefits to pain management in both general and obstetrical settings, with the most consistent benefits seen for nociceptive, acute, and postoperative pain. It has also been shown to reduce anxiety and depression and increase patient satisfaction.^24^ Specifically to obstetrics, aromatherapy has been employed in the intrapartum setting, with studies demonstrating benefits for both labor pain and anxiety.^25^

Limited data are available for use in the postpartum setting.^26,27^ Likewise, the use of massage and support persons has also shown benefit in pain management.^28–30^ More widespread use of these modalities may offer an opportunity to manage postpartum pain through nonopioid methods and more data regarding their efficacy and implementation in the postpartum setting are needed.

While there is a growing body of literature examining nurses' perspectives on pain in other areas of medicine,^11–13,31,32^ similar research in the postpartum setting is more limited. Instead, prior work in the postpartum setting has largely focused on patients' or prescribers' perspectives. This study is unique in its assessment of other care team members, namely postpartum nurses' perspectives. Importantly, this added knowledge helps to facilitate continued work to optimize pain management in the postpartum period. Future studies should continue to address and involve nursing staff as team members who play an important role in managing patient pain.

This study was conducted at a large delivery center, therefore allowing for a large sample size. Additionally, involvement of nurses in survey design and distribution improved relevance. This study is not without limitations. The single-site nature of the study as well as the lack of gender and racial–ethnic diversity among survey respondents may limit the generalizability of these findings. Additionally, survey responses may be susceptible to recall bias or social desirability bias.

## Conclusions

The results of this study provide much-needed insight into the considerations and factors affecting nursing staffs' use of opioids for pain management in the postpartum setting. We found that nurses rely on routine habits, patient preference, and patient-reported pain score when administering opioids.

Additionally, this survey demonstrated variability in training experiences as well as personal perspectives on various aspects of opioid pain management. Increased training opportunities to improve consistency and standardization of opioid administration as well as training regarding the use of nonpharmacologic options may be beneficial in postpartum pain management care.

## Supplementary Material

Supplemental data

## Abbreviations Used

ACOG: American College of Obstetricians and Gynecologists
IQR: interquartile range
MMEs: morphine milligram equivalents
PRN: *pro re nata*
REDCap: Research Electronic Data Capture
