# Supplementary material for: Nurses' Perspectives on Postpartum Pain Management
Source: Womens Health Rep (New Rochelle). 2022 Mar 4;3(1):318–25. doi: 10.1089/whr.2021.0104 (PMC8994431; doi:10.1089/whr.2021.0104)
Supplement: Supplemental data [file Suppl_Data.pdf]

Nurses' Perspectives on Postpartum Pain Management

Demographics

What is your age?

____
(years)

What is your gender?

- ◯ Male
- ◯ Female
- ◯ Prefer Not to Answer
- ◯ Other

if other, please specify

____

Are you of Hispanic, Latino, or of Spanish origin?

- ◯ Yes
- ◯ No
- ◯ Prefer Not to Answer

Which one of these groups best represents your race?

- ◯ Asian
- ◯ Black or African American
- ◯ White
- ◯ Prefer Not to Answer
- ◯ Other

if other, please specify

____

Work/Education

Which clinical location do you primarily work?

- ☐ Labor and Delivery
- ☐ Antepartum
- ☐ Postpartum
- ☐ Newborn Nursery
- ☐ Other

if other, please specify

____

On average, how many hours per month do you spend working in clinical care?

____
(hours)

On average, how many hours per month do you spend working in administration and/or teaching?

____
(hours)

How many years have you been in clinical practice?

____
(years)

How many years have you worked at Prentice?

____
(years)

What is your maximum level of education?

- ◯ Associates Degree
- ◯ Bachelor's Degree
- ◯ Master's Degree
- ◯ Doctorate
- ◯ Other

if other, please specify

____

What nursing degree do you hold?

- ◯ Associate's Degree in Nursing (ADN)
- ◯ Bachelor's of Science in Nursing (BSN)
- ◯ Master's of Science in Nursing (MSN)
- ◯ Advanced Practice Nurse (APN)
- ◯ Other

If other, please specify

____

Have you received any of the following forms of training on best practives for pain management?

- ☐ Online Module
- ☐ In-person Lectures
- ☐ Leadership Email Communications
- ☐ Colleague Mentorship
- ☐ During Coursework for Degree
- ☐ Other
- ☐ None

(choose all that apply)

if other, please specify

____

was any of the training specific to pain management in the postpartum period?

- ☐ Online Module
- ☐ In-person Lectures
- ☐ Leadership Email Communications
- ☐ Colleague Mentorship
- ☐ During Coursework for Degree
- ☐ Other

do you feel the training provided you the necessary knowledge?

- ◯ Yes
- ◯ No
- ◯ Unsure

estimate total hours of pain management training received

____
(hours)

Have you received any of the following forms of training on best practives for opoid use (ex. norco, oxycodone, tramadol, ect.) for pain management?

- ☐ Online Module
- ☐ In-person Lectures
- ☐ Leadership Email Communications
- ☐ Colleague Mentorship
- ☐ During Coursework for Degree
- ☐ Other
- ☐ None

(choose all that apply)

if other, please specify

____

was any of the training specific to pain management in the postpartum period?

- ☐ Online Module
- ☐ In-person Lectures
- ☐ Leadership Email Communications
- ☐ Colleague Mentorship
- ☐ During Coursework for Degree
- ☐ Other

do you feel the training provided you the necessary knowledge?

- ◯ Yes
- ◯ No
- ◯ Unsure

estimate total hours of opioid use for pain management training received

____
(hours)

Patient Care

Please Choose the characteristics that most influence the type and amount of pain medication you administer.

- ☐ Standard prescribing habit for all patients
- ☐ Provider preference
- ☐ Patient preference
- ☐ Patient reported pain scale
- ☐ Patient history of prescription narcotic use
- ☐ Patient comorbidities
- ☐ Patient age
- ☐ Fear of patient dissatisfaction
- ☐ Concern for potential opioid abuse
- ☐ Complexity of case
- ☐ Other

(select all that apply)

if other, please specify

____

In patients who have a vaginal delivery, what proportion of people do you think will require an opioid (ex. norco, oxycodone, tramadol, ect.) for pain management?

- ◯ None
- ◯ Few
- ◯ Half
- ◯ Most
- ◯ All

In patients who have a cesarean delivery, what proportion of people do you think will require an opioid (ex. norco, oxycodone, tramadol, ect.) for pain management?

- ◯ None
- ◯ Few
- ◯ Half
- ◯ Most
- ◯ All

Indicate your level of agreement/disagreement for each of the following statements

	Strongly Disagree	Disagree	Neutral	Agree	Strongly Agree
You should assess a patient for risk factors for opioid abuse prior to administration of an opioid (ex. norco, oxycodone, tramadol, ect).	◯	◯	◯	◯	◯
Opioids should be avoided in patients with a history of substance abuse.	◯	◯	◯	◯	◯
Vital Signs are a reliable indicator of the intensity of a patient's pain.	◯	◯	◯	◯	◯
Patients should be encouraged to endure as much pain as possible prior to using an opioid.	◯	◯	◯	◯	◯
It is important to maximize non-opioid pain medication use (ex. ibuprofen, tylenol, ect.) prior to consideration of adding an opioid.	◯	◯	◯	◯	◯
A patient's reported pain score is very important to the decision to administer an opioid.	◯	◯	◯	◯	◯

at what pain score (1 through 10) would you recommend an opioid for pain management?

◯ 1
◯ 2
◯ 3
◯ 4
◯ 5
◯ 6
◯ 7
◯ 8
◯ 9
◯ 10

How often do you recommend these forms of pain management?

	Never	Rarely	Sometimes	Often	Always
Abdominal Binders	◯	◯	◯	◯	◯
Heating/Cooling Pads	◯	◯	◯	◯	◯
Perineal Ice Packs	◯	◯	◯	◯	◯
Benzocaine Spray	◯	◯	◯	◯	◯
Lidoderm Patches	◯	◯	◯	◯	◯
Massage	◯	◯	◯	◯	◯

Aromatherapy	○	○	○	○	○
Support Person	○	○	○	○	○
Ibuprofen	○	○	○	○	○
Tylenol	○	○	○	○	○
Opioids (ex. norco, oxycodone, tramadol, ect.)	○	○	○	○	○
Other	○	○	○	○	○

if other, please specify ______________________

How helpful do you feel these forms of pain management are?

	Not Helpful	Somewhat Helpful	Very Helpful	N/A
Abdominal Binders	○	○	○	○
Heating/Cooling Pads	○	○	○	○
Perineal Ice Packs	○	○	○	○
Benzocaine Spray	○	○	○	○
Lidoderm Patches	○	○	○	○
Massage	○	○	○	○
Aromatherapy	○	○	○	○
Support Person	○	○	○	○
Ibuprofen	○	○	○	○
Tylenol	○	○	○	○
Opioids (ex. norco, oxycodone, tramadol, ect.)	○	○	○	○
Other (as specified above)	○	○	○	○

Indicate your level of agreement/disagreement for each of the following statements

	Strongly Disagree	Disagree	Neutral	Agree	Strongly Agree
Patients who lack social support have higher levels of pain.	○	○	○	○	○
Patients cared for by residents have less pain.	○	○	○	○	○
Patients cared for by private attendings often require less opioids for pain management.	○	○	○	○	○
Hispanic women are more likely to report lower levels of pain.	○	○	○	○	○
Hispanic women are more likely to decline opioid pain medication.	○	○	○	○	○

Patients with mood disorders often require more opioid pain medication.	○	○	○	○	○
Patients with pregnancy complications often require more opioids for pain management.	○	○	○	○	○
I am concerned about the effect of opioid use for postpartum pain management on breastmilk and the neonate.	○	○	○	○	○
I worry that patients will not be able to care for their neonate if they use opioids for pain management.	○	○	○	○	○
I am concerned patients' pain is being sub-optimally controlled due to under-use of opioids.	○	○	○	○	○
I am concerned patient satisfaction with postpartum care will be affected if not offered opioid for pain control.	○	○	○	○	○

Please record your level of agreement/disagreement to the following statements.

	Strongly Disagree	Disagree	Neutral	Agree	Strongly Agree
I like the autonomy that PRN opioid orders provide.	○	○	○	○	○
I feel comfortable titrating PRN opioid orders to provide appropriate pain management.	○	○	○	○	○
I feel knowledgeable about non-medication forms of pain management (ie. binders, aromatherapy, heat, ect.).	○	○	○	○	○
I feel empowered to recommend non-medication forms of pain management.	○	○	○	○	○
I would like more specific and detailed pain management protocols for postpartum pain management.	○	○	○	○	○
I would like additional training in opioid specific pain management.	○	○	○	○	○

I feel more prescriber (MD, APN, CNM) support is needed in postpartum pain management.	◯	◯	◯	◯	◯
I would like more autonomy with regards to postpartum pain management.	◯	◯	◯	◯	◯
I feel as though I have an important role to play in mitigating the opioid crisis.	◯	◯	◯	◯	◯

Discharge

When reviewing discharge information, do you discuss pain management?	◯ Yes<br>◯ No
When reviewing discharge information, do you review opioid medication disposal?	◯ Yes<br>◯ No
if yes, please specify methods of disposal that you discuss	__________________________________
How concerned are you that patients may misuse an opioid prescription after discharge?	◯ Not at all concerned<br>◯ A little concerned<br>◯ Very concerned

Comments/Concerns

What are some ways that you believe pain management could be improved in the postpartum setting?	__________________________________
Any comments and/or concerns in regards to postpartum pain management or opioid use for postpartum pain management?	__________________________________
